# Pregestational body mass index and weight gain during pregnancy associated with epidemiological variables and socio-demographic

**DOI:** 10.15446/rsap.V26n1.111293

**Published:** 2024-01-01

**Authors:** Jenniffer A. Castellanos-Garzón, Liliana Salazar-Monsalve, Antonio J. Tascon, María C. Pustovrh-Ramos

**Affiliations:** 1 JC: Biol. Ph. D. Biomedical Sciences. Faculty of Engineering, Unidad Central del Valle del Cauca. Tuluá, Colombia. Jacastellanos@uceva.edu.co Unidad Central del Valle del Cauca Faculty of Engineering Unidad Central del Valle del Cauca Tuluá Colombia Jacastellanos@uceva.edu.co; 2 LS: Physiother. Ph. D. Biological Sciences. Morphology Department, Universidad del Valle, San Fernando Campus. Cali, Colombia. liliana.salazar@correounivalle.edu.co Universidad del Valle Morphology Department Universidad del Valle Cali Colombia liliana.salazar@correounivalle.edu.co; 3 AT: MD. Clinver Research Group, Clinica Versalles. Cali, Colombia ajtascon@clinicaversalles.com.co Clinica Versalles Clinver Research Group Clinica Versalles Cali Colombia ajtascon@clinicaversalles.com.co; 4 MP: Biol. Ph. D. Biological Sciences. Morphology Department, Universidad del Valle - San Fernando Campus. Cali, Colombia. maría.pustovrh@correounivalle.edu.co Universidad del Valle Morphology Department Universidad del Valle Cali Colombia maría.pustovrh@correounivalle.edu.co

**Keywords:** Body mass index, gestational obesity, pregestational obesity, pregnancy *(source: MeSH, NLM)*, Índice de masa corporal, obesidad gestacional, obesidad pregestacional, embarazo *(fuente: DeCS, BIREME)*

## Abstract

**Objective:**

To correlate the pregestational Body Mass Index and weight gain during pregnancy with various epidemiological variables.

**Methodology:**

A cross-sectional study was carried out in a third level hospital in Cali, Colombia. Socioeconomic and anthropometric data, obstetric history and general medical history was collected from 300 pregnant women aged between 18 and 37. BMI was calculated at the beginning and end of pregnancy. Statistical analysis of multiple linear regression was performed.

**Results:**

An increased BMI at the beginning and end of gestation positively correlated with age (Coefficient = 0.156; p=0.013, Coefficient = 0.153; p=0.011), diagnosis of gestational diabetes (Coefficient = 2.264, p=0.018, Coefficient = 0.153; p=0.011) and concern about weight gain during pregnancy (Coefficient = 1.226; p=0.038, Coefficient = 1.568; p=0.004). A low BMI correlated negatively with Intrauterine Growth Restriction (Coefficient = -3.208; p=0.005). Furthermore, a higher final BMI positively correlated with a diagnosis of hypertensive disorder (Coefficient = 2.733; p<0.001) and negatively with socioeconomic status (Coefficient = 2.239; p=0.045).

**Conclusion:**

Excessive weight gain before and during pregnancy is a predictive factor in the appearance of gestational diabetes and hypertensive disorders, differentially affecting women from low socioeconomic backgrounds. While pregnancy is a critical period in a woman's life which may motivate positive lifestyle changes, excessive weight gain is still not perceived as a health problem.

## RESUMEN

**Objetivo:**

Correlacionar el Índice de Masa Corporal (IMC) pregestacional y la ganancia de peso durante el embarazo con diversas variables epidemiológicas.

**Metodología:**

Se realizó un estudio transversal en un hospital de tercer nivel de Cali, Colombia. Se recogieron datos socioeconómicos, antropométricos, antecedentes obstétricos y antecedentes médicos generales de 300 mujeres embarazadas, con edades comprendidas entre 18 y 37 años. Se calculó el IMC al inicio y al final del embarazo. Se realizó análisis estadístico de regresión lineal múltiple.

**Resultados:**

Un aumento del IMC al inicio y al final de la gestación se correlacionó positivamente con la edad (coeficiente = 0,156; p=0,013, coeficiente = 0,153; p=0,011), diagnóstico de diabetes gestacional (coeficiente = 2,264, p=0,018, coeficiente = 0,153; p=0,011) y preocupación por el aumento de peso durante el embarazo (coeficiente = 1,226; p=0,038, coeficiente = 1,568; p=0,004). Un IMC bajo se correlacionó negativamente con la restricción del crecimiento intrauterino (coeficiente = -3,208; p=0,005). Además, un IMC final más alto se correlacionó positivamente con el diagnóstico de trastorno hipertensivo (coeficiente = 2,733; p<0,001) y negativamente con el nivel socioeconómico (coeficiente = 2,239; p = 0,045).

**Conclusión:**

El aumento excesivo de peso antes y durante el embarazo es un factor predictivo en la aparición de diabetes gestacional y trastornos hipertensivos, lo que afecta diferencialmente a mujeres de estratos socioeconómicos bajos. Si bien el embarazo es un período crítico en la vida de una mujer, que puede motivar cambios positivos en el estilo de vida, el aumento excesivo de peso todavía no se percibe como un problema de salud.

Obesity is a major public health concern, known as a complex multifactorial disease. Sinse 1998 the World Health Organization (WHO) declared obesity a global epidemic 1,2 given its dramatic increase in prevalence in recent decades, particularly in the adult population in the western world and other developed countries 3. Today, obesity is also an increasingly common reality for poor and developing countries.

It is estimated that around 39% of adults worldwide have problems with excess weight and obesity 4. Obesity rates are higher in women - something which has far-reaching negative effects on reproductive health and specifically in pregnancy, a stage of life characterized by continual changes in the female body, including psycho-emotional, physiological and morphological variations which impact both her health and that of the developing fetus 5,6.

Changes in lifestyle in women of reproductive age, pointing to a higher risk pregnancy for women who begin their pregnancy overweight or obese, they gain weight risk of gestational diabetes, pre-eclampsia, eclampsia, fetal macrosomia and maternal mortality 1,7. Obesity is also associated with the future development of chronic non-communicable diseases in both the mother and child, medical situations which represent an emerging burden on the healthcare sector 8. Some research has even examined the role the uterine environment plays in the future development of obesity in the child 9. Overall, maternal obesity is associated with a three-fold higher risk of offspring obesity. Has been shown that a higher maternal prepregnancy body mass index also associat with a higher childhood total body fat mass, android/gynoid fat mass ratio, and abdominal subcutaneous and preperitoneal fat mass. Also been associated with a higher blood pressure, adverse lipid profile and higher inflammatory markers in child- hood, child- hood asthma and lead to increased risks of adiposity, and adverse cardiovascular and respiratory related outcomes in children 8.

The WHO defines nutritional status using body mass index (BMI), from which, the guidelines of the Institute of Medicine and the National Research Council recommend recording a woman's weight at the start of her pregnancy to determine total weight gain during the gestation period. In addition, they recommend that pregnancy be started with a healthy body weight, that the weight gain during pregnancy is within the recommended parameters, and after delivery there is a return to a healthy weight (10). The distinct categories of pregestational and gestational BMI help to distinguish the impact of maternal weight gain on the weight of the newborn 11, given that poor nutrition can permanently affect the function of the vital organs which determine fetal growth, and raise the risk of suffering from a number of chronic diseases throughout their life 12. Although these recommendations are taken into account in different health institutions worldwide, they must be validated in each population 10.

However, the etiology of obesity is highly complex, encompassing genetic, environmental, political and socio-economic factors, as well as cultural beliefs, social structures, physiological changes in the body's endocrine system, and epigenetic contributions across generations 9, where new relatively sedentary lifestyles, the proliferation of unhealthy and excess calorie diets, lead to the imbalance between the energy ingested and the caloric expenditure to satisfy metabolic needs 12, being foods with an industrial process or ultra-processed those that contribute the highest level of calories to consumption diets [7], making it difficult to develop effective interventions both locally and globally 1. Changing modern lifestyle habits and socioeconomic conditions may explain why weight and body mass index (BMI) are increasing worldwide 13,14. Likewise, in Colombia, according to data collected in the National Survey of the Nutritional Situation (ENSIN, 2010), 34.6% of pregnant women presented excess weight for gestational age, and in Cali, the place where this Research, a study on early fetal mortality, found that 35% of the cases presented between 2014 and 2015, 18% of pregnant women were overweight and 16.4% were obese. Therefore, it is important to identify, based on social and environmental contexts, the factors associated with weight gain in women in order to contribute to the design of preventive strategies for those overweight and obese throughout their life cycle. The objective of this study was to analyze the association between the pregestational BMI and weight gain during pregnancy in relation to epidemiological and sociodemographic factors, medical history, as well as social and cultural contexts.

## MATERIALS AND METHODOLOGY

This study was carried out at the Clinica Versalles in Cali, Colombia, was approved by the Institutional Review Committee of Human Ethics at the Universidad del Valle via Act No. 009-017 and by the Ethics Committee of the Versalles Clinic, Act No. 36-Cal-5. All participants provided written informed consent before study enrollment and received no financial contribution for their participation.

### Volunteer recruitment

A cross-sectional study was conducted with 300 women aged between 18 and 37 years with a single fetus pregnancy at full-term (between 37.0 and 41.6 weeks of gestation) who attended the delivery and caesarean section of the Versalles Clinic and had signed the informed consent form.

### Data collection

A four-part survey was conducted with all participants, collecting sociodemographic data, information on the ongoing pregnancy, anthropometric data, and obstetric and family history. The information provided by the participants was verified and complemented with the prenatal checkup record, medical history and laboratory examinations. The time taken to complete the survey was approximately 30 minutes and it was carried out by personnel trained to respond to any concerns of the participants.

### Data categorization

For the descriptive analysis of the pregnant participants, the sociodemographic component included questions about age, current pregnancy state, ethnicity, city of origin, city of residence, socioeconomic status, employment status, marital status and education level. The questions about pregnancy at the time of conducting the survey gathered data on: gestational age, gestational diabetes, hypertensive disorder in pregnancy and intrauterine growth restriction (IUGR). In the anthropometric section, the following information was recorded: most recent weight before pregnancy, height (cm), weight gain registered in prenatal checkups, weight at the time of the survey, attendance and checkups with the nutritionist. The final question was asked to establish whether women during their pregnancies had concerns related to the physical changes they had experienced during pregnancy, such as weight gain, with the answer options: *never, sometimes* and *often.*

### Body Mass Index (BMI)

BMI (BMi=weight [kg] / height [m2]) was calculated at the beginning and at the end of the pregnancy. Patients' initial nutritional status was calculated using their BMI in the first prenatal control. Participants were categorized in accordance with the following WHO BMI nutritional status classification: <18.5 = Underweight; 18.5-24.9 = Normal weight; 25.0-29.9 = Overweight; 30.0-34.9 = Obesity class I; 35.0-39.9 = Obesity class II, and 40 or more = Obesity class III. For the BMI classification at the end of pregnancy, the participants were weighed at the time of the survey. Weight gain (kg) was calculated, and the WHO classification were used to categorize the participants: initial weight BMI <20 and a weight gain of 12-18 kg; initial weight BMI between 20-24.9 and a weight gain of 10-13 kg; initial weight BMI between 25-29.9 with a gain of 7-10 Kg, and initial weight BMI >30 with a weight gain 6-7 kg. With the records obtained, participants were grouped according to final BMI as follows: a) participants with their expected final weight gain; b) those with more than the expected gain, or c) those with less than the expected gain.

### Statistical Analysis

The data was analyzed using SPSS software, version 26.0 for Windows. The results of the descriptive analysis are presented as numbers and percentages or medians and interquartile ranges (IQR). The influence on the BMI and the variables was determined by means of a multivariate linear regression analysis.

## RESULTS

### Descriptive analysis

Participants were grouped by age. Descriptive statistics indicated that the mean age was 26 years, with a standard deviation of 5.1. All participants in the study had a single pregnancy at term, that is, between 37.0 and 41.6 weeks. The mean gestational age was 39.1 weeks with a standard deviation of 1.2. The sociodemographic characteristics of the population are presented in Table 1.

**Table 1:** The sociodemographic characteristics of participants

	n	%
Age		
18-22	72	24.00
23-27	104	34.67
28-32	76	25.33
33-37	45	15.00
38-42	3	1.00
Gestational age (weeks)		
37	48	16.00
38-38.6	66	22.00
39-39.6	87	29.00
40 - 40.6	64	21.33
41	35	11.67
Ethnic group		
Mixed ethnicity	223	74.33
Afrocolombian	72	24.00
Indigenous	5	1.67
Socioeconomic status		
1	75	25.00
2	114	38.00
3	84	28.00
4	16	5.33
5	2	0.67
6	1	0.33
ND^*^	8	2.67
Employment status	0.00	
Employed	127	42.33
Employed & Studying	51	17.00
Unemployed	122	40.67
Marital status		
Married	60	20.00
In a relationship	195	65.00
Single	44	14.67
Widowed	1	0.33
Education level		
Primary incomplete	5	1.67
Primary complete	2	0.67
Secondary education complete	117	39.00
Secondary education incomplete	12	4.00
Technical studies	108	36.00
University Bachelors degree	49	16.33
Postgraduate	7	2.33

^*^ ND: No data available.

It is important to inform the reader that the socioeconomic levels in Colombia is the classification according to the payment of public services (eg, water, electricity). It is carried out mainly to collectively charge (by strata) home public services, allowing the allocation of subsidies and the collection of contributions. In this way, those with more economic capacity (strata 5 and 6) pay more for public services and contribute so that the lower strata (1 and 2) can pay their rates, strata 3 and 4 are classified as middle class.

The characteristics presented by the participants during pregnancy are shown in Table 2. None of the participants reported having had twins in previous pregnancies. Six pregnant women (2%) were diagnosed with a fetal malformation by prenatal checkup radiography, specifically, dilatation of the right kidney, a small femur, nasal hypoplasia, malformation of the feet, a narrowed pulmonary artery and an intracardiac focus on the left ventricle in the heart.

**Table 2:** Characteristics of pregnancies (n=300)

	n	%
Number of births		
1	138	46.00
2	107	35.67
3	38	12.67
4	14	4.67
5	2	0.67
8	1	0.33
Gestational diabetes		
Yes	32	10.67
No	268	89.33
Hypertensive disorder during pregnancy		
Yes	47	15.67
No	253	84.33
Intrauterine growth restriction (IUGR)		
Yes	22	7.33
No	278	92.67
Fetal malformation		
Yes	6	2.00
No	294	98.00
Nutritionist checkup		
Yes	224	74.67
No	70	23.33
ND	6	2.00
Question: Have you felt concern about weight gain or other physical changes during your pregnancy?		
Never	144	48.00
Sometimes	110	36.67
Often	44	14.67
ND*	2	0.67

^*^ ND: No data available.

The women were found to attend at least one prenatal checkup and a maximum of 12 checkups during pregnancy with a mean average of seven checkups and a standard deviation of 1.8.

### Classification according to Body Mass Index (BMI)

The calculation of participants' initial BMI was made from the weight registered in the first prenatal checkup (kg/m^2^) and the final BMI. Participants were then reclassified after this second BMI, taking into account the expected normal weight gain, and a starting BMI of 26 (kg/m^2^) was found with a standard deviation of 5.1. The minimum value was 14.6 (kg/m^2^) and the maximum value was 47.4 Kg/m^2^. The mean of the final BMI was 30 (kg/m^2^) with a standard deviation of 6.1. The minimum BMI value was 18.2 kg/ m2 and the maximum value 48.7 (kg/m^2^). The results are presented in Table 3.

**Table 3:** Classification according to BMI at the start and end of pregnancy

Starting BMI	n	%	Final weight	n	%
Underweight BMI >20 kg/m2	14	4.7	Normal range	6	42.9
			< Expected weight gain	4	28.6
			Gestational obesity	4	28.6
Normal weight BMI 20 - 24.9 kg/m2	132	44.0	Normal range	70	53.0
			Gestational obesity	56	42.4
			ND	6	4.5
Overweight BMI 25 - 29.9 kg/m2	91	30.3	Overweight	91	100.0
Obesity class I BMI >30.0 - 34.9	43	14.3	Expected weight gain	7	16.3
			> Expected weight gain	26	60.5
			< Expected weight gain	9	20.9
			ND	1	2.3
Obesity class II BMI >35.0 - 39.9	18	6.0	Expected weight gain	3	16.7
			> Expected weight gain	6	33.3
			< Expected weight gain	8	44.4
			ND	1	5.6
Obesity class III BMI >40.0	2	0.7	> Expected weight gain	1	50.0
			< Expected weight gain	1	50.0

### Correlation between BMI and epidemiological characteristics

For the multiple linear regression analysis, the initial and final BMI were considered the response variables, and the following considered predictor variables: mother's age, gestational age, ethnicity, city of residence, socioeconomic status, occupation, marital status, education level, number of prior births, gestational diabetes, hypertensive disorder in pregnancy, intrauterine growth restriction, number of prenatal checkups, and mother's concern about physical changes and weight gain during pregnancy. Independent models were made for each of the response variables.

When adjusting the multiple regression model for the initial BMI, it was found that this index increases in relation to age of the mother (Coefficient=0.156; p=0.013). In addition, a higher initial BMI correlated positively with the diagnosis for gestational diabetes (Coefficient=2.264; p=0.018), and concern about weight gain during pregnancy (Coefficient=1.226; p=0.038). In contrast, a lower BMI was recorded for pregnant women who presented IUGR (Coefficient-3,208; p=0.005) (Table 4).

**Table 4:** Variables associated with BMI at the start of pregnancy. A model of multiple linear regression with BMI as a dependent variable

Model	Non-standardized coefficients		Standardized coefficients	t	Sig.
	B	Standard Error	Beta		
(Constant)	14.419	10.313		1.398	.163
Age	.159	.064	.158	2.488	.013
Gestational age	.105	.254	.025	.415	.679
Ethnicity	.089	.697	.007	.127	.899
Resident city	-.700	.655	-.064	-1.068	.286
Socioeconomic status	2.011	1.224	.097	1.643	.101
Employment status	.023	.635	.002	.036	.971
Marital status	-.757	.844	-.053	-.897	.370
Education level	.663	.632	.064	1.050	.295
Number of births	.613	.460	.082	1.332	.184
Gestational diabetes	2.264	.948	.138	2.389	.018
Hypertensive disorder	1.529	.847	.106	1.805	.072
Intrauterine growth restriction	-3.208	1.122	-.166	-2.859	.005
Prenatal checkups	-.013	.166	-.005	-.078	.938
Number of prenatal checkups	1.299	.674	.110	1.926	.055
Concern about weight gain	1.226	.588	.119	2.084	.038

End of pregnancy BMI was found to increase with the age of the mother (Coefficient=0.153; p=0.0n). In addition, a higher BMI at the end of pregnancy positively correlated with a gestational diabetes diagnosis (Coefficient=0.153; p=0.0n), a greater concern about weight gain during pregnancy (Coefficient=1.568; p=0.004) and a diagnosis of hypertensive disorder (Coefficient=2,733; p≤0.001). In addition, a high final BMI and greater than expected weight gain was found in women from lower socioeconomic backgrounds (Coefficient=2,239; p=0.045). In contrast, however, a lower BMI correlated positively with the presence of IUGR (Coefficient-2,381; p=0.023) (Table 5).

**Table 5:** Variables associated with end of pregnancy BMI. A model of multiple linear regression with BMI as a dependent variable

Model	Non-standardized coefficients		Standardized coefficients	t	Sig.
	B	Standard Error	Beta		
(Constant)	13.325	9.434		1.412	.159
Age	.153	.059	.166	2.574	.011
Gestational age	.269	.233	.070	1.158	.248
Ethnicity	.458	.647	.042	.708	.479
Resident city	-.542	.607	-.054	-.893	.372
Socioconomic status	2.239	1.111	.121	2.016	.045
Employment status	.026	.590	.003	.045	.964
Marital status	-.973	.780	-.074	-1246	.214
Education level	.397	.587	.042	.676	.500
Number of births	-.193	.436	-.028	-.443	.658
Gestational diabetes	1.679	.885	.111	1.898	.059
Hypertensive disorder	2.733	.770	.211	3.549	.000
Intrauterine growth restriction	-2.381	1.040	-.135	-2.288	.023
Prenatal checkups	-.090	.154	-.035	-.584	.560
Number of prenatal checkups	.575	.637	.052	.902	.368
Concern about weight gain	1.568	.544	.167	2.882	.004

## DISCUSSION

The principal objective of this study was to correlate pregestational BMI and weight gain during pregnancy with epidemiological variables. The findings indicate that 44% of pregnant women started their pregnancy within a BMI within the normal range. However, although this was the prevalent classification (44%), more than half the pregnant women started their pregnancy in unhealthy nutritional states, whether underweight, overweight, or obese. Almost a third (30.3%) of the participants were overweight and 21% were obese. This data concurs with the majority of studies from western countries where the prevalence of obesity was found to be 30% in 2015 4. When comparing our data with studies on European populations, similar prevalence levels of maternal overweight have been reported in Norway (20%), and in other European countries 4,15,16. Although the percentages of overweight and obese pregnant women in this study are high, they are not comparable to those found in the United States, where 64% of women of reproductive age are overweight and 35% are classified as obese 4.

Being overweight or obese prior to pregnancy, as well as experiencing excessive weight gain during pregnancy, are negative factors associated with complications for both the pregnant woman and her offspring 1,7,17. Excess weight makes women high-risk patients as these conditions are associated with complications in pregnancy such as gestational diabetes, hypertension, pre-eclampsia, instrumented birth, impaired fetal growth, among other complications 18-20,19-23.

Our research showed that increased BMI in early pregnancy, classified as pre-pregnancy obesity, correlates positively with maternal age and the presence of gestational diabetes, data supported in the literature where it is reported that prevalence of obesity in pregnant women has occurred concurrently with an increase in gestational diabetes, associated with adverse metabolic adaptations in the mother. These include increased risks to compromise fetal and newborn survival and health [19]. These findings coincide with research conducted in Argentina over four years 24, in which it was determined that women starting pregnancy aged 30 or more (49.30%) and with a BMI equal to or greater than 27 kg/m^2^ were a combined risk factor for developing gestational diabetes.

Arterial hypertension during pregnancy is a serious public health problem and is the main cause of maternal and fetal morbimortality in many world countries. In our study, we found that an increased final BMI also correlated positively with the risk of hypertensive disorder in pregnancy. It is known that most cases of hypertension during pregnancy occur in healthy women, therefore, it is important to establish the epidemiological characteristics that can influence the development of this pathology. However, several studies indicate a relationship between hypertension in pregnancy and increased body mass index, explained by several mechanisms: insulin resistance and hyperinsulinemia, increased adrenergic activity and aldosterone concentrations, sodium retention, water and alteration. of endothelial function, through molecules such as leptin and adiponectin and genetic factors 25.

Several studies report that this disorder is associated with increased maternal BMI, such as those conducted in the Netherlands, Norway and Spain. Gaillard *et al* (2013) 26 conducted a prospective study in the Netherlands with 6,959 patients and found maternal obesity to be associated with an increased risk of gestational hyper-tension (OR 6.31 (95% CI 4.30, 9.26)) and gestational diabetes (OR 6.28 (95% CI 3.01, 13.06)). In addition, they reported an increase in maternal complications when excess weight was acquired during the first trimester of pregnancy 24. In Norway, similar findings were published by Huagen *et al* (2014) 15 in a prospective study involving 56,101 pregnant women. A Spanish study following 500 pregnant women, found that those who gained weight above recommended levels had gestational hypertension (3.4%) and gestational diabetes (1.4%) as the most frequent pathologies. 18-20.

Intrauterine growth restriction (IUGR) is one of the reported complications associated with unhealthy maternal weight. In this study, a low initial BMI correlated positively with an IUGR diagnosis, a condition reported in the literature as occurring when there is a structural and functional deficiency of the placenta that can be caused by inadequate placental angiogenesis associated with a deficiency in essential fatty acids required for fetoplacental growth and development 27,28. Essential fatty acids are known to be fundamental and involved in every stage of pregnancy by supporting cell growth and development, cell signaling, and modulation of other critical aspects of structural and functional processes 27. On the contrary, in a study carried out in Romania in 2012, a higher incidence of IUGR was observed in patients with obesity 28. Therefore, it is possible to suggest that an unfavorable uterine environment generated by an initial high or low BMI is associated with IUGR, a pathology considered an important cause of fetal, perinatal and neonatal morbidity and mortality 29-31.

Numerous experimental and epidemiological studies have demonstrated that nutritional changes in the maternal pregestational and gestational stages have a significant impact on maternal and fetal health, and child development. Globalization has led to cultural changes in food consumption by flooding the world market with high-calorie products, including those high in fructose and cheap, refined fats which lead to an increase in the consumption of carbohydrates consumed compared to more traditional diets 2,14. The impact that these processed products have on the population is difficult to quantify. However, their contribution to the increase in overweight and obese people is evident. Factors such as education, income level, physical activity, smoking, and alcohol consumption, however, are also related to the percentage of calorie intake from different macronutrients 14. In this study, a high final BMI was linked to low socioeconomic status, findings that concur with research by Ohlsson and Manjer 14 indicating that sociodemographic factors and lifestyle habits affect body weight and body composition. Furthermore, as reported by Manrique (2017) 18, the inverse relationship between socioeconomic status and obesity in women could be mediated by the fact that women in developed societies have developed dieting habits, perform more physical activity and can adopt a healthy diet, which typically involves a greater economic investment, conditions that are not so common in those from lower socioeconomic backgrounds 18. This data also coincides with research carried out in the Nether-lands, in which a lower family income was associated with a higher risk of maternal obesity (p<0.05) 26. These are some ways in which sociodemographic factors can be predictors of gestational weight gain. There is a complex interaction between biological, psychological and social contextual factors that influence the amount of weight a woman gains in pregnancy 32, as indicated in research by McLaren 33, in a review of 333 studies. This review found a close relationship between obesity and economic and sociocultural factors, revealing a greater prevalence of this condition in women from developing countries.

Although the implications on fetal and neonatal health were not examined in this study, the literature highlights the consequences of obesity and how these extend beyond the women themselves, impacting their offspring 1,7,17. Newborns of mothers obese during pregnancy are more likely to have greater complications in the perinatal period 18. At the same time, an obesogenic environment during the preconception and gestational periods will increase the child's risk of developing obesity over the long term 18. Similarly, children of obese women have a higher incidence of congenital defects of the central nervous system, larger blood vessels, abdominal wall and intestine, defects that may be caused by an insufficient absorption or insufficient distribution of essential nutrients such as folic acid, or an effect of the incipient hyperglycemia caused by insulin resistance at the time of organogenesis 18. Although this study did not find a positive correlation between pregestational or gestational obesity with malformations in the fetus, it found that 2% of fetuses presented one of the following diagnoses; right kidney dilation, small femur, nasal hypoplasia, malformed feet, a narrowed pulmonary artery or an intracardiac focus on the left ventricle. In relation to these findings, there are studies that indicate that excess maternal body weight before pregnancy increases the absolute risk of many ultrasound-detected, adverse outcomes in the fetus 34. Furthermore, maternal factors such as diet, obesity and endocrine dysfunction can modulate the expression and activity of placental fatty acid distribution and, therefore, affect fetoplacental growth and development 27.

Finally, the participants were asked if they were concerned about physical changes or weight gain during pregnancy. A positive association was found between the participants with an affirmative answer and an increased BMI both at the beginning and at the end of their pregnancy. Participants expressing this concern were expected to be become more cautious in their food consumption and this would be reflected in a final BMI within the recommended range. However, this was not the case. As Rodríguez-Guzmán *et al*^35^ explain, although BMI is an objective indicator of weight, body image scales are subjective. These researchers in their study on self-perception of body image and its correlation with BMI, found that overweight or obese women frequently perceive themselves as being of normal weight. A similar study carried out in Argentina in 2015 36, reported that 15% of young women in pregnancy underestimated their body image size, where the majority were overweight and obese. Our work also found a significant association between concern about body weight and advanced maternal age. A study by Borelli *et al,* also found a significant association between these two factors (x2 = 12.639; gl=4; p=0.013) 36. With regard to the self-perception of body image and weight gain, it can be concluded that, although these variables may be a concern during pregnancy, overweight or obese women tend to have a distorted perception of their body size in relation to reality.

Excessive weight gain before and during pregnancy is a predictive factor in the appearance of gestational diabetes and hypertensive disorders, both of which differentially affect women from low socioeconomic backgrounds. While pregnancy is a critical period in a woman's life and can be the motivation to make positive lifestyle changes, excess weight is not yet perceived as a significant health problem. These results and those of previous studies conducted on this subject worldwide highlight the impact of maternal obesity beyond the intrauterine and newborn phase of life to the increased risks of generating long-term health complications into adult life. In addition, there is a strong associated sociocultural component in that, via an overweight mother, an overweight family is likely born through transmission of behaviors and eating habits ♣
